# Community Racial and Ethnic Representation Among Physicians in US Internal Medicine Residency Programs

**DOI:** 10.1001/jamanetworkopen.2024.57310

**Published:** 2025-01-30

**Authors:** Jung G. Kim, Elle Lett, Christy K. Boscardin, Karen E. Hauer, Isabel L. Chen, Mark C. Henderson, Sean O. Hogan, Kenji Yamazaki, Jesse Burk-Rafel, Tonya Fancher, Mytien Nguyen, Eric S. Holmboe, William McDade, Dowin H. Boatright

**Affiliations:** 1 Ronald O. Perelman Department of Emergency Medicine, New York University Langone Health, New York; 2 Institute for Innovations in Medical Education, New York, New York; 3 Perelman School of Medicine at the University of Pennsylvania, Philadelphia; 4 Department of Medicine, University of California, San Francisco; 5 Kaiser Permanente Bernard J. Tyson School of Medicine, Pasadena, California; 6 Department of Internal Medicine, University of California, Davis, Sacramento; 7 Accreditation Council for Graduate Medical Education, Chicago, Illinois; 8 Intealth, Philadelphia, Pennsylvania

## Abstract

**Importance:**

Increasing underrepresented in medicine (URIM) physicians among historically underserved communities helps reduce health disparities. The concordance of URIM physicians with their communities improves access to care, particularly for American Indian and Alaska Native, Black, and Hispanic or Latinx individuals.

**Objectives:**

To explore county-level racial and ethnic representation of US internal medicine (IM) residents, examine racial and ethnic concordance between residents and their communities, and assess whether representation varies by presence of academic institutions or underserved settings.

**Design, Setting, and Participants:**

This retrospective cross-sectional study collected data from the Association of American Medical Colleges, Accreditation Council for Graduate Medical Education (ACGME), Area Health Resources Files, and US Department of Education data on ACGME-accredited US IM residency programs and their associated county populations. Self-reported racial and ethnic data from 2018 for 4848 residents in 393 IM programs in 205 counties were used. Data were analyzed between February 15 and September 20, 2024.

**Exposure:**

County-level presence for academic health centers (AHCs), minority-serving institutions (MSIs), health professional shortage areas (HPSAs), and rurality.

**Main Outcomes and Measures:**

Main outcomes were representation quotients (RQs) or the ratio of the proportion of IM residents and their concordant county-level racial and ethnic populations. Quantile linear regression models on median representation were used to identify the association with URIM, Asian, and White residents by US Census division and county-level AHCs, MSIs, HPSAs, and rurality.

**Results:**

Among 4848 residents, 4 (0.08%) self-identified as American Indian or Alaskan Native, 1709 (35.3%) as Asian, 289 (6.0%) as Black, 211 (4.4%) as Hispanic or Latinx, 2 (0.04%) as Native Hawaiian or Other Pacific Islander, and 2633 (54.3%) as White. A total of 761 (15.7%) were classified as URIM. Among URIM groups, American Indian and Alaska Native (mean [SE] RQ, 0.00 [0.04]), Black (mean [SE] RQ, 0.09 [0.20]), Hispanic and Latinx (mean [SE] RQ, 0.00 [0.04]), and Native Hawaiian and other Pacific Islander (mean [SE] RQ, 0.00 [0.26]) residents were grossly underrepresented compared with their training sites’ county-level representation. Fifty-one of 205 counties (24.8%) with IM programs had no URIM residents. Black and Hispanic or Latinx residents had higher representation in counties with more MSIs (mean [SD] RQ, 0.19 [0.24]; *P* = .04; mean [SD] RQ, 0.15 [0.04]; *P* < .001, respectively), and Hispanic or Latinx residents were less represented in counties with more AHCs (mean [SD] RQ, 0.00 [0.06]; *P* < .001). Asian residents had lower RQs in counties with more MSIs (mean [SD] RQ, 6.00 [0.65]; *P* < .001), and White residents had higher representation in counties with greater presence of AHCs (mean [SD] RQ, 0.77 [0.04]; *P* = .007).

**Conclusions and Relevance:**

In this cross-sectional study, URIM IM residents remained underrepresented compared with their program’s county populations. These findings should inform racial and ethnic diversity policies to address the continuing underrepresentation among graduate medical education physicians, which adversely impacts the care of historically underserved communities.

## Key Points

**Question:**

What is the racial and ethnic concordance of internal medicine residents compared with their local communities, and does that vary by the presence of academic institutions or underserved settings?

**Findings:**

This cross-sectional study found that among 4848 internal medicine residents training across 205 counties, historically underrepresented in medicine individuals remained underrepresented compared with their training program’s local populations. County clustering and presence of academic health centers and minority-serving institutions were associated with differing county-level racial and ethnic representation.

**Meaning:**

These findings suggest that institutional characteristics may affect continued racial and ethnic underrepresentation of internal medicine residents, ultimately hampering concordant representation of historically underserved communities.

## Teaser

This cross-sectional study examines the racial and ethnic makeup of accredited US internal medicine residency programs and county-level concordance of the resident population relative to the racial and ethnic makeup of the local population.

## Introduction

A strategy for closing racial and ethnic disparities is increasing community representation within the physician workforce.^1,2^ Increasing the number of physicians from underrepresented in medicine (URIM) and historically underserved communities reduces health disparities affecting Black, Latinx, and Native American individuals, who are disproportionately affected by chronic illnesses, including type 2 diabetes and hypertension.^3,4^ Concordance between URIM physicians and their respective communities improves access to care, responsiveness to community needs, and equitable treatment.^1,2,5,6,7,8,9,10,11,12,13^

Physician workforce planning to address health care disparities aims to bolster “racial and ethnic populations underrepresented relative to their numbers in the general population.”^6,14,15^ For example, educational pathway programs at minority-serving institutions (MSIs) increase physician workforce diversity through focused recruitment of URIM students.^16,17^ The role of institutional factors to improve racial and ethnic representation remains understudied, including the presence of MSIs and training in academic health centers (AHCs), where URIM physicians remain underrepresented.^10^

Studies have examined racial and ethnic representation trends across health professions, but less is known about concordant representation of resident physicians relative to the local communities served during training.^18,19,20,21^ Graduate medical education (GME) is a pivotal training period in which residents are embedded into communities, developing their clinical skills to address health disparities.^10,22^ GME workforce planning policies have relied on county-level indicators, including health professional shortage areas (HPSAs) and degree of rurality to identify workforce gaps.^23,24^ Few studies assess the intersection of academic institutional factors and the extent of GME URIM representation relative to local communities.

This national study examines the racial and ethnic makeup of residents in accredited US internal medicine (IM) residency programs and county-level racial and ethnic concordance of the resident population relative to the makeup of the local population. We selected IM residency programs because of their varied training settings, inclusion of both primary care and subspecialty career pathways, and degree of racial and ethnic underrepresention.^21,25,26^ We also investigated whether the presence of academic institutions or underserved communities was associated with representation of major racial and ethnic groups.

## Methods

The study followed the Strengthening the Reporting of Observational Studies in Epidemiology (STROBE) reporting guideline. The New York University institutional review board designated this study as not human participant research, and informed consent was therefore not required.

### Data Sources

This retrospective cross-sectional analysis examined integrated data from the Association of American Medical Colleges (AAMC), Accreditation Council for Graduate Medical Education (ACGME), Area Health Resources Files (AHRF), and the US Department of Education for residents training in ACGME-accredited US IM programs in 2018. The AAMC tracks the self-reported race and ethnicity of all resident physicians; we examined available aggregated race and/or ethnic groups in IM residencies for American Indian or Alaska Native, Asian, Black, Hispanic or Latinx, Native Hawaiian or Other Pacific Islander, and White (non-Hispanic), as well as residents’ GME training program setting (AHC county presence). URIM was categorized using the AAMC definition.^14^ Our analysis excluded residents classified as multiracial (≥2 races) because Hispanic or Latinx residents are not included in multiracial categories within the AHRF. We also excluded residents born outside the US because non–US citizens are not classified by race and ethnicity in the AAMC dataset. Age and gender data were not available for analysis.

These data were linked to ACGME data to identify a program’s location by Federal Information Processing Standards (FIPS), which then were linked to the 2020-2021 AHRF by FIPS (state and county), a publicly available dataset published by the Health Resources and Services Administration’s Bureau of Health Workforce. AHRF data include county-level HPSAs, rurality, and census of racial and ethnic populations in the AHRF matched to AAMC race and ethnicity categories.^27^ To identify the extent of pathway programs at the county level, the dataset was then linked by FIPS to the US Department of Education’s Office of Postsecondary Education directory to identify associations with MSIs.^16^ MSIs are composed of Alaska Native and Native Hawaiian–serving institutions, Asian American and Native American and Pacific Islander–serving institutions, Hispanic-serving institutions, Native American–serving nontribal institutions, predominantly Black institutions, historically Black colleges and universities (HBCUs), and tribal colleges and universities.

### Statistical Analysis

Analyses were conducted between February 15, and September 20, 2024. Concordance between the racial and ethnic makeup of IM residents and the general population geographically proximate at the US county level was assessed using the representation quotient (RQ) method.^19,28^ The RQ calculates the ratio of the proportion of IM residents from each racial and ethnic group among programs within their county relative to racial and ethnic representation in the county population.

An RQ of 1.0 indicates that the race and ethnicity of residents is equally represented in the community or county. Values less than 1.0 indicate a group is underrepresented, and values greater than 1.0 indicate overrepresentation. For example, a value of 0.1 indicates that county-wide residency program representation of a resident’s race and ethnicity is one-tenth that of the community representation; a value near 0 indicates nearly no representation of a resident’s race and ethnicity in their training community. We calculated the RQs for each racial and ethnic group and examined their frequency, central tendency, and variation across all counties and by US Census divisions. Wilcoxon rank sum tests were performed to determine associations of RQs for each racial and ethnic group by the 9 US Census divisions (Pacific, Mountain, Central-Northwest, Central-Northeast, Central-Southwest, Central-Southeast, Atlantic-South, Atlantic-Middle, and New England), presence of AHC or MSI, HPSA designation, and rurality (rural-urban continuum code >3).

To determine whether county-level median RQ values for URIM, Asian, and White IM residents were associated with US Census division, presence of academic institutions (number of AHCs and MSIs in the county), and health workforce planning indicators (county designated as HPSA or rural), we performed multivariable bootstrapped (20×) quantile linear regression models at the 50th percentile of the RQ. Quantile regression also accounted for the relative low magnitude of URIM representation nationally because we could not model disaggregated racial and ethnic groups for American Indian or Alaska Native, Hispanic or Latinx, and Native Hawaiian or other Pacific Islander residents due to their low reported frequencies. We also performed sensitivity analyses using multivariable bootstrapped quantile linear regression models at the 75th percentile of the RQ to account for county-level factors associated with greater racial and ethnic representation.

Stata, version 18.0 (StataCorp LLC) was used to conduct these analyses. To visualize county-level variation across the US, we performed geospatial analyses for URIM, Asian, and White RQs. R software, version 4.3.3 (R Foundation) packages ggplot and usmap were used to analyze and visualize representation at the county level across the US. A 2-sided *P* < .05 was considered statistically significant.

## Results

In 393 ACGME-accredited US IM programs across 205 counties, 4848 residents self-reported their race and ethnicity as American Indian or Alaska Native (4 [0.08%]), Asian (1709 [35.25%]), Black (289 [5.96%]), Hispanic or Latinx (211 [4.35%]), Native Hawaiian or other Pacific Islander (2 [0.04%]), and White (2633 [54.31%]). Of these, 761 (15.70%) were classified as URIM. Among counties with IM residency programs, 117 (57.1%) had AHCs (eFigure 1 in Supplement 1), 112 (54.6%) had MSIs (eFigure 2 in Supplement 1), 195 (95.1%) were in HPSAs, and 39 (19.0%) were designated as rural. Of the US Census divisions, 19 counties (9.3%) were located in the Pacific region, 9 (4.4%) in the Mountain region, 14 (6.8%) in the Central-Northwest, 33 (16.1%) in the Central-Northeast, 24 (11.7%) in the Central-Southwest, 12 (5.9%) in the Central-Southeast, 43 (21.0%) in the Atlantic-South, 37 (18.1%) in the Atlantic-Middle, and 14 (6.8%) in New England (Table 1).

**Table 1.** Internal Medicine Residency Program Resident- and County-Level Characteristics

Characteristic	No. (%) (N = 205 counties and 4848 residents)
Resident race and ethnicity	
American Indian and Alaska Native	4 (0.08)
Asian	1709 (35.3)
Black	289 (6.0)
Hispanic and Latinx	211 (4.4)
Native Hawaiian and other Pacific Islander	2 (0.04)
White	2633 (54.3)
Underrepresented in medicine^14^	761 (15.7)
County-level race and ethnicity of population	
American Indian and Alaska Native	1 615 359 (1.0)
Asian	13 437 234 (8.5)
Black	27 314 766 (17.4)
Hispanic and Latinx	38 510 943 (24.5)
Native Hawaiian and other Pacific Islander	473 684 (0.3)
White	109 744 283 (69.8)
Underrepresented in medicine^14^	67 914 752 (43.2)
US Census divisions of counties	
Pacific	19 (9.3)
Mountain	9 (4.4)
Central-Northwest	14 (6.8)
Central-Northeast	33 (16.1)
Central-Southwest	24 (11.7)
Central-Southeast	12 (5.9)
Atlantic-South	43 (21.0)
Atlantic-Middle	37 (18.1)
New England	14 (6.8)
Health professional shortage area counties	195 (95.1)
Rural counties	39 (19.0)
Counties with academic health centers	117 (57.1)
Counties with minority-serving institutions	112 (54.6)

### RQs by Racial and Ethnic Groups

The median (SE) RQs by race and ethnicity for IM residents relative to their training program’s county racial and ethnic representation are reported here (Table 2). Among URIM groups, American Indian and Alaska Native, Black, Hispanic and Latinx, and Native Hawaiian and other Pacific Islander residents were grossly underrepresented compared with their training sites’ county-level representation.

**Table 2.** Median (95% CI) Representation Quotients by Race and Ethnicity for Overall County Level and by County Characteristics

Location	Median (95% CI) representation quotient						
	American Indian and Alaska Native	Asian	Black	Hispanic and Latinx	Native Hawaiian or other Pacific Islander	White	URIM
Overall (n = 205 counties)	0.00 (0.00-0.00)	7.44 (6.19-8.07)	0.09 (0.00-0.19)	0.00 (0.00-0.00)	0.00 (0.00-0.00)	0.73 (0.66-0.78)	0.36 (0.27-0.42)
Pacific (n = 19)	0.00 (0.00-0.00)	3.91 (1.62-5.67)^a^	0.00 (0.00-0.06)^b^	0.07 (0.00-0.18)	0.00 (0.00-0.00)^b^	0.68 (0.44-0.79)	0.43 (0.23-0.52)
Mountain (n = 9)	0.00 (0.00-0.00)	5.45 (0.0-7.04)^b^	0.37 (0.00-2.57)	0.00 (0.00-0.38)	0.00 (0.00-0.00)	0.73 (0.45-1.10)	0.25 (0.16-0.53)
Central-Northwest (n = 14)	0.00 (0.00-0.00)	6.42 (1.68-13.6)	0.00 (0.00-0.25)	0.00 (0.00-0.00)	0.00 (0.00-0.00)	0.92 (0.66-1.11))^b^	0.21 (0.00-0.40)
Central-Northeast (n = 33)	0.00 (0.00-0.00)	8.92 (7.48-14.10)^a^	0.16 (0.00-0.50)	0.00 (0.00-0.00)^a^	0.00 (0.00-0.00)	0.73 (0.56-0.84)	0.39 (0.18-0.63)
Central-Southwest (n = 24)	0.00 (0.00-0.00)	7.28 (4.22-14.10)	0.00 (0.0-0.66)	0.00 (0.00-0.2)	0.00 (0.00-0.00)	0.77 (0.41-0.89)	0.25 (0.1-0.69)
Central-Southeast (n = 12)	0.00 (0.00-0.00)	12.1 (6.4-17.0)^b^	0.05 (0.00-0.34)	0.00 (0.00-0.00)	0.00 (0.00-0.00)	0.94 (0.72-1.42)^c^	0.11 (0.0-0.41)
Atlantic-South (n = 43)	0.00 (0.00-0.00)	7.76 (4.28-10.39)	0.22 (0.00-0.4)	0.00 (0.00-0.35)	0.00 (0.00-0.00)	0.81 (0.59-0.99)	0.40 (0.23-0.54)
Atlantic-Middle	0.00 (0.00-0.00)	7.6 (6.0-9.16)	0.23 (0.00-0.40)	0.00 (0.00-0.26)	0.00 (0.00-0.00)	0.57 (0.45-0.65)^c^	0.42 (0.29-0.62)
New England (n = 14)	0.00 (0.00-0.00)	5.72 (3.51-8.62)	0.00 (0.00-0.25)	0.17 (0.00-0.36)	0.00 (0.00-0.00)	0.70 (0.52-0.81)	0.47 (0.15-0.89)
Health professional shortage area (n = 195)	0.00 (0.00-0.00)	7.46 (6.16-8.11)	0.11 (0.00-0.20)	0.00 (0.00-0.00)^c^	0.00 (0.00-0.00)	0.72 (0.66-0.78)	0.37 (0.28-0.43)
Rural (n = 39)	0.00 (0.00-0.00)	16.90 (8.10-29.8)^c^	0.00 (0.00-0.00)^a^	0.00 (0.00-0.00)^c^	0.00 (0.00-0.00)	0.76 (0.66-0.84)	0.00 (0.00-0.47)^b^
Academic health center (n = 117)	0.00 (0.00-0.00)	6.65 (5.50-8.00)	0.19 (0.07-0.25)	0.00 (0.00-0.00)^b^	0.00 (0.00-0.00)	0.77 (0.70-0.82)^a^	0.37 (0.28-0.44)
Minority-serving institution (n = 112)	0.00 (0.00-0.00)	6.01 (4.75-7.3)^c^	0.19 (0.09-0.28)^c^	0.15 (0.00-0.22)^c^	0.00 (0.00-0.00)	0.70 (0.61-0.81)	0.35 (0.27-0.43)

Abbreviation: URIM, underrepresented in medicine.

^a^ *P* < .01.

^b^ *P* < .05.

^c^ *P* < .001.

For American Indian and Alaska Native residents, the mean (SE) RQ was 0.00 (0.04), with the Central-Northeast US Census division reporting the largest mean (SE) representation at 0.00 (0.24). No representation was found in the Mountain, Central-Northwest, Central-Southeast, Atlantic-Middle, Atlantic-South, and New England US Census Divisions.

For Asian residents, the mean (SE) RQ was 7.40 (1.20), with the Central-Southeast US Census division reporting the highest at a mean (SE) of 12.10 (3.60). Asian residents had statistically significantly higher representation in the Central-Northeast (mean [SE] RQ, 8.90 [3.60]; *P* < .001) and in the Central-Southeast (mean [SE] RQ, 12.10 [3.60]; *P* = .01) US Census divisions but lower RQs in the Pacific (mean [SE] RQ, 3.90 [0.95]; *P* = .01) and Mountain (mean [SE] RQ, 5.50 [1.50]; *P* = .04) US Census divisions. Asian residents also had higher RQs in rural counties (mean [SE] RQ, 16.90 [5.00]; *P* < .001).

For Black residents, the mean (SE) RQ was 0.09 (0.20), with the highest in the Mountain division (mean [SE] RQ, 0.37 [0.38]). Black residents had lower RQs in the Pacific US Census division (mean [SE] RQ, 0.13 [0.22]; *P* = .04) and in rural counties (mean [SE] RQ, 0.00 [0.19]; *P* = .002).

For Hispanic and Latinx residents, the mean (SE) RQ was 0.00 (0.04), with the highest in the New England division (mean [SE] RQ, 0.17 [0.27]). Hispanic and Latinx residents had lower RQs in the Central-Northeast division (mean [SE] RQ, 0.00 [0.06]; *P* < .001) and in rural counties (mean [SE] RQ, 0.00 [0.04]; *P* < .001).

For Native Hawaiian and other Pacific Islander residents, the mean (SE) RQ was 0.00 (0.26), with the highest in the Atlantic-South division (mean [SE] RQ, 0.00 [1.26]) and the lowest in the Pacific division (mean [SE] RQ, 0.00 [0.29]; *P* = .048). Native Hawaiian and other Pacific Islander residents were not represented in the Mountain, Central-Northwest, Central-Northeast, Central-Southwest, Central-Southeast, Atlantic-Middle, and New England US Census divisions. These residents also had lower RQs in HPSA counties (mean [SE] RQ, 0.00 [5.40]; *P* < .001).

For White (non-Hispanic) residents, the mean (SE) RQ was 0.73 (0.03), with the highest in the Central-Southeast division (mean [SE] RQ, 0.94 [0.22]). Statistically significant higher RQs were observed in the Central-Northwest (mean [SE] RQ, 0.90 [0.06]; *P* = .046) and Central-Southeast (mean [SE] RQ, 0.77 [0.13]; *P* = .004) US Census divisions and lower RQs in the Atlantic-Middle division (mean [SE] RQ, 0.57 [0.05]; *P* = .001).

Across all counties, 51 counties (24.9%) had no URIM resident representation for an estimated mean (SD) of 85 383 (111 012) URIM individuals in those counties. Sixteen (7.8%) had no Asian resident representation for a mean (SD) of 11 215 (20 487) Asian residents across the associated counties. Five (2.4%) had no White resident representation for a mean (SD) of 203 652 (166 003) White residents in the counties.

### RQs by Institutional Factors

Black and Hispanic or Latinx residents had higher representation in counties with more MSIs (mean [SD] RQ, 0.19 [0.24]; *P* = .04; mean [SD] RQ, 0.15 [0.04]; *P* < .001, respectively), and Hispanic or Latinx residents were less represented in counties with more AHCs (mean [SD] RQ, 0.00 [0.06]; *P* < .001). Asian residents had lower RQs in counties with more MSIs (mean [SD] RQ, 6.00 [0.65]; *P* < .001), and White residents had higher representation in counties with a greater presence of AHCs (mean [SD] RQ, 0.77 [0.04]; *P* = .007) (Table 2).

In the adjusted multivariable quantile analyses (Table 3), compared with nonrural counties, rural counties were associated with differing median RQs (coefficient, −0.251; 95% CI, −0.500 to −0.002; *P* = .049) for URIM residents. A greater presence of AHCs was associated with higher than the median RQ for White residents (coefficient, 0.003; 95% CI, 0.001-0.000; *P* < .001), whereas a greater presence of MSIs was associated with lower than the White resident median RQ (coefficient, −0.018; 95% CI, −0.031 to 0.005; *P* = .006).

**Table 3.** Multivariable Estimates of County-Level Characteristics by Change in Median

County characteristic	URIM		Asian		White	
	Adjusted coefficient (95% CI)	*P* value	Adjusted coefficient (95% CI)	*P* value	Adjusted coefficient (95% CI)	*P* value
US Census division						
Pacific	−0.033 (−0.369 to 0.302)	.85	−1.697 (−6.071 to 2.677)	.45	0.098 (0.118 to 0.313)	.37
Mountain	−0.234 (−0.544 to 0.076)	.14	−0.703 (−6.195 to 4.788)	.80	0.242 (0.227 to 0.71)	.31
Central-Northwest	−0.247 (−0.572 to 0.079)	.14	−2.954 (−12.290 to 6.381)	.53	0.174 (0.072 to 0.420)	.17
Central–Northeast	−0.070 (−0.426 to 0.286)	.70	3.193 (−1.077 to 7.463)	.14	0.104 (0.164 to 0.372)	.45
Central-Southwest	−0.101 (−0.545 to 0.342)	.65	0.965 (−2.900 to 4.830)	.62	0.149 (0.13 to 0.429)	.29
Central–Southeast	−0.248 (−0.582 to 0.086)	.14	5.335 (−2.779 to 13.450)	.20	0.344 (0.058 to 0.746)	.09
Atlantic-South	−0.094 (−0.410 to 0.222)	.56	1.235 (−3.429 to 5.899)	.60	0.149 (0.098 to 0.395)	.24
Atlantic–Middle	−0.038 (−0.381 to 0.306)	.83	1.896 (−1.945 to 5.738)	.33	−0.144 (−0.351 to 0.062)	.17
New England	1.0 [Reference]	NA	1.0 [Reference]	NA	1.0 [Reference]	NA
Health professional shortage area	−0.003 (−0.842 to 0.836)	.99	0.938 (−5.732 to 7.607)	.79	0.002 (0.285 to 0.290)	.99
Rural	−0.251 (−0.500 to −0.002)	.049	10.345 (−1.585 to 22.276)	.09	−0.085 (0.259 to 0.089)	.34
Academic health center	0.000 (−0.002 to 0.001)	.80	−0.019 (−0.044 to 0.005)	.12	0.003 (0.001 to 0.004)	<.001
Minority-serving institution	−0.003 (−0.014 to 0.007)	.53	−0.007 (−0.108 to 0.094)	.89	−0.018 (−0.031 to 0.005)	.006

Abbreviations: NA, not applicable; URIM, underrepresented in medicine.

Sensitivity analyses for Asian and URIM residents set at higher representation levels (at the 75th percentile) showed these residents to be less likely to be in counties with a greater presence of AHCs. A greater presence of AHCs in the county was associated with RQs lower than the 75th percentile for URIM residents (coefficient, −0.002; 95% CI, −0.005 to −0.000; *P* = .04) and Asian residents (coefficient, −0.038; 95% CI, −0.066 to −0.009; *P* = .009). Compared with nonrural counties, rural counties were associated with an RQ higher than the 75th percentile for Asian residents (coefficient, 23.830; 95% CI, 16.440-31.220; *P* = .001). Census divisions associated with an RQ greater than the 75th percentile for White residents were found in the Mountain (coefficient, 0.324; 95% CI, 0.077-0.571; *P* = .01) and Central-Northwest (coefficient, 0.410; 95% CI, 0.126-0.688; *P* = .005) US Census divisions (eTable in Supplement 1).

The Figure illustrates the RQ gradients nationally, with darker shades indicating the county is more represented by URIM, Asian, and White racial and ethnic groups in US IM residency programs. eFigures 1 and 2 in Supplement 1 illustrate the number of AHCs and MSIs, with darker shades indicating greater presence of each respective organization.

**Figure.**
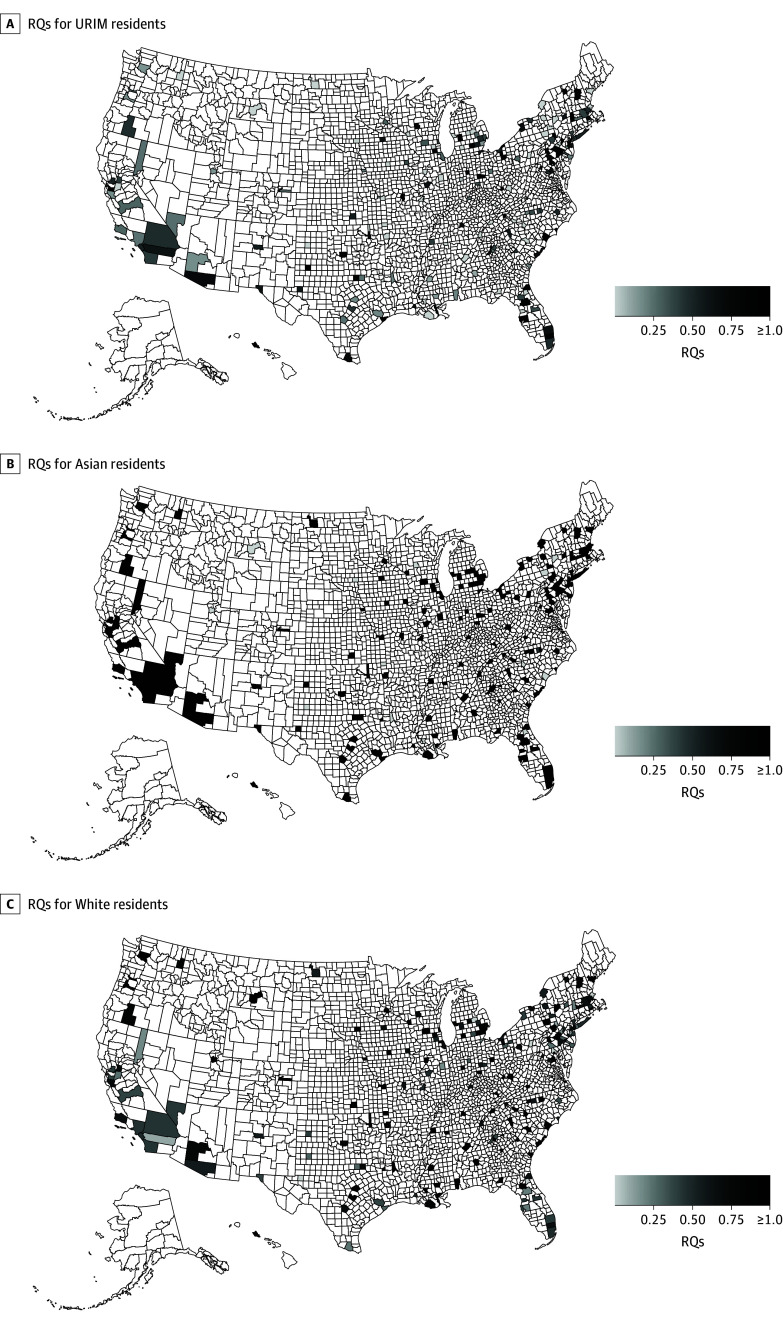
Representation Quotients (RQs) for Underrepresented in Medicine (URIM), Asian, and White Residents in US Accreditation Council of Graduate Medical Education–Accredited Internal Medicine Programs RQs are illustrated by shade intensity based on a scale of 0 to 1.00 or greater. An RQ that is 1.00 or greater indicates the racial or ethnic group is fully represented among US-accredited internal medicine residency program. Unshaded counties illustrate the county has no internal medicine residency program.

## Discussion

This national study reports the racial and ethnic representation of ACGME-accredited US IM residency programs and the persistent underrepresentation of specific racial and ethnic groups among resident physicians relative to local communities. Overall, we found continuing and low representation for American Indian and Alaska Native, Black, Hispanic and Latinx, and Native Hawaiian and other Pacific Islander groups among IM residents compared with their county communities.^18,19^ Although 1 in 3 individuals in the US is Black or Hispanic or Latinx, we found that nearly one-quarter of counties did not have any URIM resident representation in IM residency programs, representing more than 85 000 individuals living in counties with no racial and ethnic GME representation. We found that a greater presence of AHCs in the county was associated with higher White resident representation; in contrast, a higher county presence of MSIs was associated with greater URIM representation.

The lack of representation by American Indian and Alaska Native, Black, Hispanic and Latinx, and Native Hawaiian and other Pacific Islander residents is a stark reminder that equitable representation remains an unrealized aspirational goal. Because AHCs and HPSAs typically serve underserved patients, including many from the Black and Hispanic and Latinx communities, URIM resident representation and their clustering and nonrandom distribution require further examination.^29,30^ In contrast, the overall overrepresentation of Asian residents relative to their local and national population, particularly in rural counties, highlights the need to disaggregate racial and ethnic data on Asian subgroups, who encompass a wide range of ethnicities.^31^ Lack of disaggregated data within available datasets limits the ability to draw clearer conclusions about the associations of specific racial and ethnic groups in GME.

We found no association between HPSAs and higher URIM representation in IM GME. The adjusted analyses did not yield higher than median or 75th percentile URIM representation compared with Asian and White residents in counties designated as HPSAs. Although Hispanic and Latinx individuals account for approximately 25% of our sample’s US population, Hispanic and Latinx residents were less represented in HPSAs, underscoring concerns about underrepresentation of this fast-growing community.^20^ HPSA is an important needs-based designation informing policymakers about primary care access and understanding health disparities.^32^ HPSA counties include populations vulnerable to poorer health outcomes, highlighting the need for a representative workforce to close these disparities.^33,34,35^ The HPSA designation is a key criteria for awarding National Health Services Corps scholarships and federal loan repayment programs totaling more than $300 million, supporting URIM individuals pursuing careers in underserved communities.^36,37,38^ Calibrating the HPSA designation to prioritize, track, and increase racial and ethnic community representation is essential for reducing health disparities. Additional research is critical to understand the relationship of URIM representation in GME to the care of local underserved populations within HPSA counties.

Adjusted multivariable analyses revealed greater representation for White residents in counties with more AHCs. AHCs serve as the gateway to more competitive medical subspecialties and fellowships and as a base for producing nationally funded biomedical research.^39,40^ URIM representation among faculty in academic medicine remains low (<10%), and although URIM medical school enrollment has increased slightly, the vast majority of incoming residents lack URIM faculty mentor representation.^41,42^ Lack of diversity in academic medicine hinders efforts to increase physician research-scientist representation and in clinical trials to benefit underserved populations.^1,43^ Ultimately, lack of community representation within AHCs may stunt scientific advances and limit care for underserved populations.

Higher Black and Hispanic and Latinx representation was associated with increasing county presence of MSIs, suggesting that educational pathway programs may improve community racial and ethnic representation in IM GME. HBCUs have been a critical pathway for increasing the representation of Black students interested in medicine.^44^ Furthermore, a workforce projection that simulated keeping closed historically Black medical schools open over the past century produced an approximately 30% increase in Black physicians.^45^ For Hispanic and Latinx students, more than half of the universities in the University of California system are Hispanic-serving institutions, having demonstrated high enrollments from students with Hispanic and Latinx heritage.^46^ Our study suggests that the higher presence of MSIs is associated with county-level resident representation within Black and Hispanic and Latinx communities, calling for continuing support for MSIs. Highlighting the pivotal role of MSIs, which have a track record of increasing URIM representation, is essential to workforce diversity across the academic medicine pillars of education, research, and clinical practice.^39,45^

### Limitations

This study has several limitations. We studied a single GME specialty and an available cross-sectional dataset with county-level characteristics. Studying other specialties is needed, including family medicine, which boasts higher racial and ethnic and rural diversity, and tertiary care–based specialties with historically low racial and ethnic diversity.^47^ Longitudinal databases examining physician racial and ethnic diversity are needed to understand the access to health professions training for URIM individuals and whether increased diversity improves clinical outcomes for disenfranchised communities.^48^ The AHRF is widely used for health workforce planning, but county-level analysis may overlook nuances more visible in a program’s hospital referral region and/or service area. The recent US Supreme Court decision banning affirmative action also highlights the continuing need to examine how to increase the representation of URIM individuals in the physician workforce and to study the impact of such federal-level decisions on the representation of URIM resident physicians in communities that most need them.^49,50,51,52^ Additionally, we must examine individuals represented by multiple races and non–US citizen physicians, who play a pivotal role for improving care access yet are largely unstudied. Lastly, the URIM designation serves as a proxy for other important measures, including socioeconomic status and first generation in higher education, that strongly influence an individual’s opportunities to pursue medicine and specialty choice.

## Conclusions

In this cross-sectional study of IM residents, there was significant and continuing underrepresentation of specific racial and ethnic groups, particularly in AHCs. We found geographic clustering of specific racial groups and MSIs, which could inform efforts on how to increase URIM representation. These findings improve our understanding of racial and ethnic representation of resident physicians in their communities, which potentially impacts the ability to close health disparities in historically underserved populations.

While national workforce policies recognize the importance of workforce diversification, our results highlight a greater urgency to address these persistent gaps.^53,54,55^ Amplified efforts are needed to integrate stakeholders across physician workforce planning and training oversight constituencies to ensure resources are available that maintain workforce equity standards. Furthermore, integrating these constituents with health professions pathway programs and community-based efforts will help ensure physician training programs are socially accountable and work to address health inequities that continue to affect the US population.^11,24,56,57^

## Notes

Supplement 1. **eFigure 1.** Number of Academic Health Centers in Counties with US ACGME-Accredited Internal Medicine Programs **eFigure 2.** Number of Minority Serving Institutions in Counties with US ACGME-Accredited Internal Medicine Programs **eTable.** Sensitivity Analyses - Multivariable Quantile Estimates of County-Level Characteristics by Change in 75th Percentile for URiM, Asian, and White Internal Medicine Residents Representation Quotients

Supplement 2.
Data Sharing Statement

## References

[1] Smedley BD, Stith AY, Nelson AR, eds; Institute of Medicine (US) Committee on Understanding and Eliminating Racial and Ethnic Disparities in Health Care. Unequal Treatment: Confronting Racial and Ethnic Disparities in Health Care. National Academies Press; 2003. Accessed June 2, 2021. https://www.ncbi.nlm.nih.gov/books/NBK220358/25032386

[2] Butler PD, Fowler JC, Meer E, Rosen IM, Reyes IM, Berns JS. A blueprint for increasing ethnic and racial diversity in U.S. residency training programs. Acad Med. 2022;97(11):1632-1636. doi:10.1097/ACM.0000000000004847 35857407

[3] Centers for Disease Control and Prevention. Interactive atlas of heart disease and stroke tables. Accessed May 22, 2024. https://nccd.cdc.gov/DHDSPAtlas/Reports.aspx

[4] Centers for Disease Control and Prevention. National diabetes statistics report. May 21, 2024. Accessed May 22, 2024. https://www.cdc.gov/diabetes/php/data-research/index.html

[5] McCauley L, Phillips RL, Meisnere M, Robinson SK, eds; Committee on Implementing High-Quality Primary Care, Board on Health Care Services, Health and Medicine Division, National Academies of Sciences, Engineering, and Medicine. *Implementing High-Quality Primary Care: Rebuilding the Foundation of Health Care.* National Academies Press; 2021. 34251766

[6] Betancourt JR. Eliminating racial and ethnic disparities in health care: what is the role of academic medicine? Acad Med. 2006;81(9):788-792. doi:10.1097/00001888-200609000-00004 16936481

[7] Cooper-Patrick L, Gallo JJ, Gonzales JJ, . Race, gender, and partnership in the patient-physician relationship. JAMA. 1999;282(6):583-589. doi:10.1001/jama.282.6.583 10450723

[8] Komaromy M, Grumbach K, Drake M, . The role of black and Hispanic physicians in providing health care for underserved populations. N Engl J Med. 1996;334(20):1305-1310. doi:10.1056/NEJM199605163342006 8609949

[9] Burkhardt J, DesJardins S, Gruppen L. Diversity of the physician workforce: specialty choice decisions during medical school. PLoS One. 2021;16(11):e0259434. doi:10.1371/journal.pone.0259434 34735513 PMC8568153

[10] Gonzaga AMR, Appiah-Pippim J, Onumah CM, Yialamas MA. A framework for inclusive graduate medical education recruitment strategies: meeting the ACGME standard for a diverse and inclusive workforce. Acad Med. 2020;95(5):710-716. doi:10.1097/ACM.0000000000003073 31702694

[11] Snyder JE, Upton RD, Hassett TC, Lee H, Nouri Z, Dill M. Black representation in the primary care physician workforce and its association with population life expectancy and mortality rates in the US. JAMA Netw Open. 2023;6(4):e236687. doi:10.1001/jamanetworkopen.2023.6687 37058307 PMC10105312

[12] Thompson HS, Manning M, Mitchell J, . Factors associated with racial/ethnic group-based medical mistrust and perspectives on COVID-19 vaccine trial participation and vaccine uptake in the US. JAMA Netw Open. 2021;4(5):e2111629. doi:10.1001/jamanetworkopen.2021.11629 34042990 PMC8160590

[13] Loeb S, Ravenell JE, Gomez SL, . The effect of racial concordance on patient trust in online videos about prostate cancer: a randomized clinical trial. JAMA Netw Open. 2023;6(7):e2324395. doi:10.1001/jamanetworkopen.2023.24395 37466938 PMC10357333

[14] Association of American Colleges. Underrepresented in medicine definition. Accessed June 28, 2024. https://www.aamc.org/what-we-do/equity-diversity-inclusion/underrepresented-in-medicine

[15] Association of American Medical Colleges. Advancing diversity, equity, and inclusion in medical education. Accessed December 16, 2024. https://www.aamc.org/about-us/equity-diversity-inclusion/advancing-diversity-equity-and-inclusion-medical-education

[16] Rutgers Graduate School of Medicine. CMSI—a brief history of MSIs. Accessed June 10, 2024. https://cmsi.gse.rutgers.edu/content/brief-history-msis

[17] US Department of the Interior. Minority serving institutions program. July 1, 2015. Accessed July 7, 2024. https://www.doi.gov/pmb/eeo/doi-minority-serving-institutions-program

[18] Salsberg E, Richwine C, Westergaard S, . Estimation and comparison of current and future racial/ethnic representation in the US health care workforce. JAMA Netw Open. 2021;4(3):e213789. doi:10.1001/jamanetworkopen.2021.3789 33787910 PMC8013814

[19] Lett E, Murdock HM, Orji WU, Aysola J, Sebro R. Trends in racial/ethnic representation among US medical students. JAMA Netw Open. 2019;2(9):e1910490. doi:10.1001/jamanetworkopen.2019.10490 31483469 PMC6727686

[20] Baker O, Horvitz-Lennon M, Yu H. Racial and ethnic concordance between National Health Service Corps clinicians and underserved populations. JAMA Netw Open. 2024;7(3):e242961. doi:10.1001/jamanetworkopen.2024.2961 38506809 PMC10955390

[21] Liao J, Nishath T, Thevuthasan S, . Race/ethnicity trends among U.S. internal medicine residency applicants and matriculants: a cross-sectional study. Ann Intern Med. 2022;175(4):611-614. doi:10.7326/M21-3287 35073150

[22] Asch DA, Nicholson S, Srinivas S, Herrin J, Epstein AJ. Evaluating obstetrical residency programs using patient outcomes. JAMA. 2009;302(12):1277-1283. doi:10.1001/jama.2009.1356 19773562

[23] Blanchard J, Petterson S, Bazemore A, Watkins K, Mullan F. Characteristics and distribution of graduate medical education training sites: are we missing opportunities to meet U.S. health workforce needs? Acad Med. 2016;91(10):1416-1422. doi:10.1097/ACM.0000000000001184 27028032

[24] Chen C, Petterson S, Phillips RL, Mullan F, Bazemore A, O’Donnell SD. Toward graduate medical education (GME) accountability: measuring the outcomes of GME institutions. Acad Med. 2013;88(9):1267-1280. doi:10.1097/ACM.0b013e31829a3ce9 23752037 PMC3761381

[25] ACP Online. History of internal medicine. Accessed July 7, 2024. https://www.acponline.org/about-acp/about-internal-medicine/career-paths/medical-student-career-path/history-of-internal-medicine

[26] Association of American Medical Colleges. Diversity in medicine: facts and figures 2019. Accessed July 11, 2024. https://www.aamc.org/data-reports/workforce/report/diversity-medicine-facts-and-figures-2019

[27] Health Resources and Services Administration. HRSA data warehouse—area health resource files. 2017. Accessed January 8, 2025. https://datawarehouse.hrsa.gov/topics/ahrf.aspx

[28] Persad-Paisley EM, Kazal FH, Shamshad A, . Applying representation quotient methodology to racial, ethnic, and gender trends of applicants and matriculants to urology residency programs from 2010-2018. Urology. 2023;172:25-32. doi:10.1016/j.urology.2022.09.032 36402268

[29] Henderson MC, Kizer KW, Kravitz RL. Academic health centers and Medicaid: advance or retreat? Acad Med. 2018;93(10):1450-1453. doi:10.1097/ACM.0000000000002295 29794521

[30] Chen AH, Ghaly MA. Medicaid as a driver of health equity. JAMA. 2022;328(11):1051-1052. doi:10.1001/jama.2022.14911 36125484

[31] Equitable Growth. How data disaggregation matters for Asian Americans and Pacific Islanders. December 14, 2016. Accessed July 9, 2024. https://equitablegrowth.org/how-data-disaggregation-matters-for-asian-americans-and-pacific-islanders/

[32] Bureau of Health Workforce. What is shortage designation? Accessed July 22, 2024. https://bhw.hrsa.gov/workforce-shortage-areas/shortage-designation

[33] Streeter RA, Snyder JE, Kepley H, Stahl AL, Li T, Washko MM. The geographic alignment of primary care health professional shortage areas with markers for social determinants of health. PLoS One. 2020;15(4):e0231443. doi:10.1371/journal.pone.0231443 32330143 PMC7182224

[34] Singh GK, Lin CCC. Area deprivation and inequalities in health and health care outcomes. Ann Intern Med. 2019;171(2):131-132. doi:10.7326/M19-1510 31261382

[35] Bennett K, Phillips J, Teevan B. Closing the gap: finding and encouraging physicians who will care for the underserved. Virtual Mentor. 2009;11(5):390-398. 23195179 10.1001/virtualmentor.2009.11.5.pfor1-0905

[36] Criteria for designation of mental health professional shortage areas–PHS: final rule. Fed Regist. 1992;57(14):2473-2480. 10116398

[37] Lee RC. Current approaches to shortage area designation. J Rural Health. 1991;7(4)(suppl):437-450. doi:10.1111/j.1748-0361.1991.tb01085.x 10116034

[38] Graham Center. The National Health Service Corps at 50 years. Accessed July 22, 2024. https://www.graham-center.org/content/brand/rgc/publications-reports/publications/one-pagers/the-national-health-service-corps-50-years.html

[39] Association of American Medical Colleges. *Handbook of Academic Medicine: How Medical Schools and Teaching Hospitals Work*. Association of American Medical Colleges; 2013.

[40] Santhosh L, Babik JM. Diversity in the pulmonary and critical care medicine pipeline: trends in gender, race, and ethnicity among applicants and fellows. ATS Sch. 2020;1(2):152-160. doi:10.34197/ats-scholar.2019-0024IN 33870279 PMC8043294

[41] Ogunwole SM, Dill M, Jones K, Golden SH. Trends in internal medicine faculty by sex and race/ethnicity, 1980-2018. JAMA Netw Open. 2020;3(9):e2015205. doi:10.1001/jamanetworkopen.2020.15205 32870313 PMC7489836

[42] Association of American Medical Colleges. New AAMC data on diversity in medical school enrollment in. 2023. Accessed July 12, 2024. https://www.aamc.org/news/press-releases/new-aamc-data-diversity-medical-school-enrollment-2023

[43] National Institute on Minority Health and Health Disparities. Strategic plan 2021-2025. May 26, 2021. Accessed May 26, 2021. https://nimhd.nih.gov/about/strategic-plan/index.html

[44] The Century Foundation. The role of HBCUs in ensuring a diverse health care workforce. October 26, 2023. Accessed July 22, 2024. https://tcf.org/content/commentary/the-role-of-hbcus-in-ensuring-a-diverse-health-care-workforce/

[45] Campbell KM, Corral I, Infante Linares JL, Tumin D. Projected estimates of African American medical graduates of closed historically Black medical schools. JAMA Netw Open. 2020;3(8):e2015220. doi:10.1001/jamanetworkopen.2020.15220 32816033 PMC7441360

[46] University of California. Minority-serving institutions. Accessed July 7, 2024. https://diversity.universityofcalifornia.edu/actions/minority-serving-institutions.html

[47] Goodfellow A, Ulloa JG, Dowling PT, . Predictors of primary care physician practice location in underserved urban or rural areas in the United States: a systematic literature review. Acad Med. 2016;91(9):1313-1321. doi:10.1097/ACM.0000000000001203 27119328 PMC5007145

[48] Hinton I, Howell J, Merwin E, . The educational pipeline for health care professionals: understanding the source of racial differences. J Hum Resour. 2010;45(1):116-156. doi:10.3368/jhr.45.1.116

[49] Saha S. After affirmative action—working toward equitable representation in medicine. N Engl J Med. 2023;389(19):1817-1821. doi:10.1056/NEJMms2308319 37937784

[50] Henderson MC, Aminololama-Shakeri S. Options for building a diverse health care workforce. JAMA. 2024;331(4):357-358. doi:10.1001/jama.2023.23397 38261054

[51] Affirmative action ends: how Supreme Court ruling impacts medical schools & the health care workforce. Accessed July 9, 2024. https://www.ama-assn.org/medical-students/medical-school-life/affirmative-action-ends-how-supreme-court-ruling-impacts

[52] Henderson MC, Fancher TL, Murin S. Holistic admissions at UC Davis—journey toward equity. JAMA. 2023;330(11):1037-1038. doi:10.1001/jama.2023.15872 37578801

[53] American Medical Association. AMA organizational strategic plan to advance health equity: 2024-2025. American Medical Association. June 27, 2024. Accessed December 7, 2024. https://www.ama-assn.org/system/files/ama-equity-strategic-plan-2024-2025.pdf

[54] Accreditation Council for Graduate Medical Education. Diversity, equity, and inclusion. Accessed July 16, 2024. https://www.acgme.org/initiatives/diversity-equity-and-inclusion/10.1016/j.wneu.2024.03.007 38458250

[55] Association of American Medical Colleges. Importance of diversity in health care. Accessed July 16, 2024. https://www.aamc.org/about-us/mission-areas/medical-education/my-story-matters

[56] Phillips RL Jr, George BC, Holmboe ES, Bazemore AW, Westfall JM, Bitton A. Measuring graduate medical education outcomes to honor the social contract. Acad Med. 2022;97(5):643-648. doi:10.1097/ACM.0000000000004592 35020616 PMC9028305

[57] Pittman P, Chen C, Erikson C, . Health workforce for health equity. Med Care. 2021;59(suppl 5):S405-S408. doi:10.1097/MLR.0000000000001609 34524235 PMC8428843

