# Supplementary material for: Community Racial and Ethnic Representation Among Physicians in US Internal Medicine Residency Programs
Source: JAMA Netw Open. 2025 Jan 30;8(1):e2457310. doi: 10.1001/jamanetworkopen.2024.57310 (PMC11783195; doi:10.1001/jamanetworkopen.2024.57310)
Supplement: Supplement 1. — eFigure 1. Number of Academic Health Centers in Counties with US ACGME-Accredited Internal Medicine Programs eFigure 2. Number of Minority Serving Institutions in Counties with US ACGME-Accredited Internal Medicine Programs eTable. Sensitivity Analyses - Multivariable Quantile Estimates of County-Level Characteristics by Change in 75th Percentile for URiM, Asian, and White Internal Medicine Residents Representation Quotients [file jamanetwopen-e2457310-s001.pdf]

Supplementary Online Content

Kim JG, Lett E, Boscardin CK, et al. Community racial and ethnic representation among physicians in US internal medicine residency programs. *JAMA Netw Open*. 2025;8(1):e2457310. doi:10.1001/jamanetworkopen.2024.57310

eFigure 1. Number of Academic Health Centers in Counties with US ACGME-Accredited Internal Medicine Programs

eFigure 2. Number of Minority Serving Institutions in Counties with US ACGME-Accredited Internal Medicine Programs

eTable. Sensitivity Analyses - Multivariable Quantile Estimates of County-Level Characteristics by Change in 75th Percentile for URiM, Asian, and White Internal Medicine Residents Representation Quotients

This supplementary material has been provided by the authors to give readers additional information about their work.

© 2025 Kim JG et al. *JAMA Network Open*.

eFigure 1: Number of Academic Health Centers in Counties with US ACGME-accredited Internal Medicine Programs

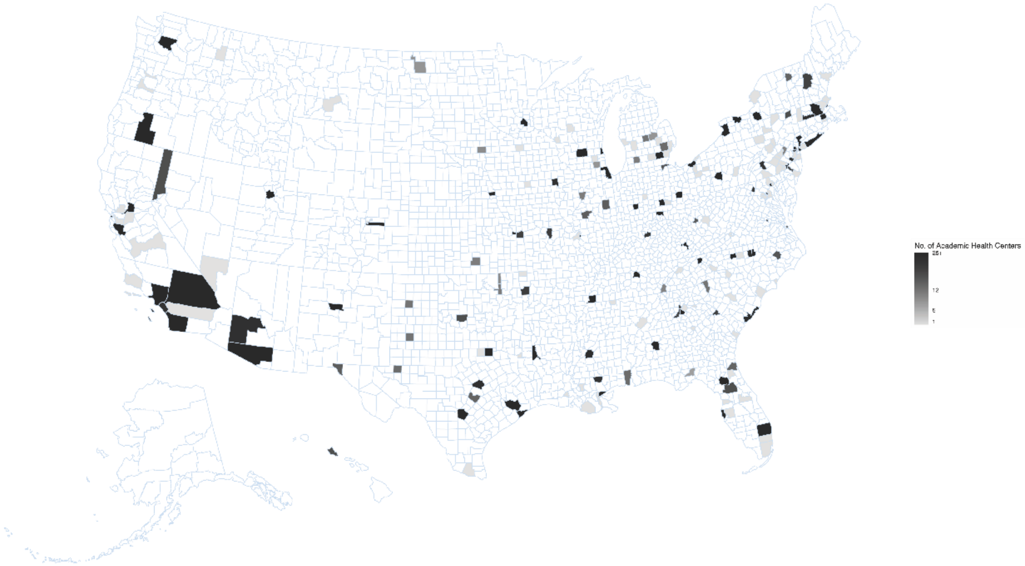

© 2025 Kim JG et al. *JAMA Network Open*.

eFigure 2: Number of Minority Serving Institutions in Counties with US ACGME-accredited Internal Medicine Programs

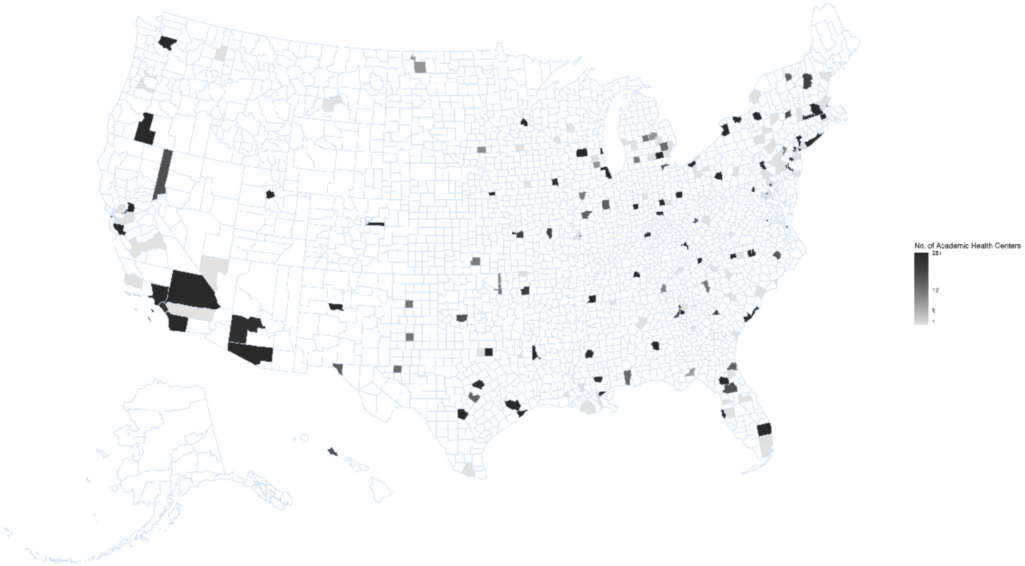

© 2025 Kim JG et al. *JAMA Network Open*.

eTable1: Sensitivity Analyses - Multivariable quantile estimates of county-level characteristics by change in 75th Percentile for URiM, Asian, and White internal medicine residents representation quotients

	URiM		Asian		White	
**County Characteristic**	Adjusted Coefficient	p-value	Adjusted Coefficient	p-value	Adjusted Coefficient	p-value
**Census Division:**						
Pacific	-0.157 (-0.849 - 0.534)	0.654	-4.710 (-10.377 - 0.956)	0.103	0.098 (-0.211 - 0.407)	0.532
Mountain	-0.417 (-1.061 - 0.226)	0.202	-2.361 (-11.479 - 6.756)	0.610	0.324 (0.077 - 0.571)	0.010
Central - Northwest	-0.266 (-0.909- .377)	0.415	1.654 (-18.654 - 21.963)	0.873	0.407 (0.126 - 0.688)	0.005
Central - Northeast	0.303 (-0.566 - 1.174)	0.492	5.188 (-13.731 - 24.108)	0.589	0.173 (0.0113 - 0.335)	0.036
Central - Southwest	-0.0680 (-0.646 -0.511)	0.817	1.413 (-6.111 - 8.938)	0.711	0.203 (-0.559 - 0.967)	0.599
Central - Southeast	-0.486 (-1.249 -0.276)	0.210	6.0420 (- 5.342 - 17.426)	0.296	0.656 (-0.434 - 1.747)	0.024
Atlantic - South	-0.0544 (- 0.644 - .536)	0.856	2.339 (-3.250 - 7.928)	0.410	0.324 (0.07 - 0.578)	0.013
Atlantic - Mid	0.0188 (-0.526 - 0.564)	0.946	1.649 (-3.575 - 6.873)	0.534	-0.043 (-0.279 - 0.193)	0.720
New England	Reference					
**Health Professions Shortage Area**	0.0641 (-0.822 - 0.951)	0.887	3.340 (-6.553 - 13.233)	0.506	-0.091 (- 0.425 - 0.244)	0.595
**Rural**	0.099 (-0.934 - 1.132)	0.850	23.833 (16.442 - 31.224)	0.001	-0.055 (- 0.268 - 0.158)	0.611
**Academic Health Center**	-0.002 (-0.005 - -0.001)	0.037	-0.0380 (-0.067 - -0.009)	0.009	0.002 (-.001 - .00541)	0.102
**Minority Serving Institution**	-0.004 (-0.019 - 0.012)	0.625	0.0189 (-0.268 - 0.306)	0.897	-0.01 (-0.02 - 0.003)	0.158

© 2025 Kim JG et al. *JAMA Network Open*.
