# Supplementary material for: Community Racial and Ethnic Representation Among Physicians in US Internal Medicine Residency Programs
Source: JAMA Netw Open. 2025 Jan 30;8(1):e2457310. doi: 10.1001/jamanetworkopen.2024.57310 (PMC11783195; doi:10.1001/jamanetworkopen.2024.57310)
Supplement: Supplement 2. — Data Sharing Statement [file jamanetwopen-e2457310-s002.pdf]

Data Sharing Statement

Kim. Community Racial and Ethnic Representation Among Physicians in US Internal Medicine Residency Programs. *JAMA Netw Open*. Published January 30, 2025.
doi:10.1001/jamanetworkopen.2024.57310

Data
Data available: No

Additional Information
Explanation for why data not available: A data use agreement with the AAMC prevents us from sharing individual level data
